# Phonological development in American Sign Language-signing children: Insights from pseudosign repetition tasks

**DOI:** 10.3389/fpsyg.2022.921047

**Published:** 2022-09-08

**Authors:** Shengyun Gu, Deborah Chen Pichler, L. Viola Kozak, Diane Lillo-Martin

**Affiliations:** ^1^Department of Linguistics, University of Connecticut, Storrs, CT, United States; ^2^Department of Linguistics, School of Language, Education, and Culture, Gallaudet University, Washington, DC, United States

**Keywords:** American Sign Language (ASL), pseudosign, child language acquisition, modality, working memory, phonological complexity, non-word repetition task, phonological development

## Abstract

In this study, we conducted a pseudosign (nonce sign) repetition task with 22 children (mean age: 6;04) acquiring American Sign Language (ASL) as a first language (L1) from deaf parents. Thirty-nine pseudosigns with varying complexity were developed and organized into eight categories depending on number of hands, number of simultaneous movement types, and number of movement sequences. Pseudosigns also varied in handshape complexity. The children’s performance on the ASL pseudosign task improved with age, displaying relatively accurate (re)production of location and orientation, but much less accurate handshape and movement, a finding in line with real sign productions for both L1 and L2 signers. Handshapes with higher complexity were correlated with lower accuracy in the handshape parameter. We found main effects of sequential and simultaneous movement combinations on overall performance. Items with no movement sequence were produced with higher overall accuracy than those with a movement sequence. Items with two simultaneous movement types or a single movement type were produced with higher overall accuracy than those with three simultaneous movement types. Finally, number of hands did not affect the overall accuracy. Remarkably, movement sequences impose processing constraints on signing children whereas complex hands (two hands) and two simultaneous movement types do not significantly lower accuracy, indicating a capacity for processing multiple simultaneous components in signs. Spoken languages, in contrast, manifest greater complexity in temporal length. Hearing children’s pseudoword repetition still displays high levels of accuracy on disyllabic words, with complexity effects affecting only longer multisyllabic words. We conclude that the pseudosign repetition task is an informative tool for studies of signing children’s phonological development and that sheds light on potential modality effects for phonological development.

## Introduction

Early investigations of the sub-lexical structure, or phonology, of sign languages, characterized the form of a sign in terms of four primary ‘parameters’: handshape, location, movement, and orientation. More recent sign phonological theories have recognized that while the concept of sign parameters is useful, more detailed analyses at the feature level can lead to greater understanding of the ways that sign phonology is organized. These developments have also contributed to a greater understanding of *complexity* in sign language phonology.

For sign languages, lexical and morphological complexities often take the form of simultaneously combined elements, rather than the sequential combinations more typical in spoken languages. This might be related to the fact that sequential memory coding is enhanced in the processing of spoken languages, while spatial memory is superior in the processing of sign languages (for a review, see Giezen, 2021). Sign languages take advantage of this difference by building complexity in primarily monosyllabic units, in which multiple components of information are simultaneously expressed, rather than employing sequences of syllables.

In this study, we ask whether this difference in phonological complexity of sign languages versus spoken languages impacts sign language development. Our data come from analysis of pseudosigns (nonce or non-word signs) reproduced by 4- to 8-year-old native signers of American Sign Language (ASL). The pseudosigns are categorized into those with greater sequential complexity, e.g., containing a sequential movement, and those with greater simultaneous complexity, e.g., involving two hands, layered movement types, or more complex handshapes. We find that, indeed, pseudosigns with a sequential movement are reproduced less accurately than those without sequential movement. On the other hand, signs with two simultaneous movement types are not produced less accurately; only when the complexity level reaches three simultaneous movement types does accuracy decrease. We also find that two-handed pseudosigns are not reproduced less accurately than one-handed pseudosigns, and in this study handshape complexity only relates to the accuracy of handshape reproduction, not overall accuracy of the sign.

In the rest of this introduction section, we provide readers with relevant background information about sign language phonology and sign phonological complexity, previous studies of sign language phonological development, and previous studies using the non-word repetition technique with both spoken and signed languages.

### Sign language phonology

**FIGURE 1:**
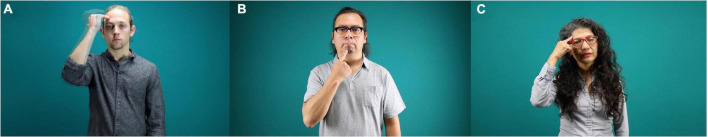
Minimal pairs in American Sign Language (ASL) (figures reproduced with permission from ASL Signbank; Hochgesang et al., 2021). **(A)** KNOWbb; **(B)** DISAPPOINT; and **(C)** THINK.

Early linguistic analyses of sign languages (Stokoe, 1960; Battison, 1978) described signs in terms of four main formational components: the configuration of the hand(s) (or handshape), the location on the body or in space in which the sign is made, the movement of the arm/hand/fingers, and the orientation of the hands (e.g., palms facing the signer, or the signer’s ipsilateral or contralateral side). Specification of the values for each of these manual ‘parameters’^1^ allows for the characterization of individual signs, capturing the possibility of minimal pairs that differ in the value of a single such parameter. For example, the signs KNOWbb^2^ and THINK (Figures 1A,C) share the same location, movement, and orientation, but differ in handshape (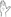 vs. 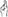), while DISAPPOINT and THINK (Figures 1B,C) share the same handshape, movement, and orientation, but differ in location (chin vs. forehead).

Today there are many theoretical models of sign language phonology, but they all start with the basic observation that values for these four parameters need to be specified to identify a sign. However, it is also clear that while signs can be decomposed into parameters, the parameters themselves are complex and can be viewed in terms of phonological features (see Section “Scoring” for descriptions of the features that we adopted for the current study). For example, the 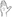 handshape of KNOWbb (Figure 1A) can be described in terms of its selected fingers (all fingers selected), joint position (selected fingers extended), and thumb position (extended). Several models have been proposed to account for the possible patterns observed for hand configurations (Sandler, 1989; Corina and Sandler, 1993; van der Hulst, 1993; van der Kooij, 2002; Sandler and Lillo-Martin, 2006), and some models have also adopted more complex representations for other parameters (movement, location, and orientation) (Brentari, 1998, 2019).

While modern approaches to sign language phonology have progressed well beyond simple parameter-based sign descriptions, the notion of parameters continues to play a large role in psycholinguistics and language acquisition. For that reason, the current project uses both parameter-based and feature-based approaches to compare different types of potential phonological complexity for signs, as well as phonological complexity between signed and spoken languages.

### Phonological complexity in sign languages

Phonological complexity of individual signs can be defined in various ways (Mann et al., 2010; Ortega and Morgan, 2015; Brentari, 2019; Morgan et al., 2019; van der Hulst and van der Kooij, 2021). For example, some signs use one hand (e.g., the three signs illustrated in Figure 1), while others use both hands (e.g., ALL-DAY and ANNOTATE). The use of two hands is potentially more complex than the use of one hand only, as it requires additional information to be specified in the sign’s lexical entry.

Another way to assess phonological complexity is by considering the complexity of individual parameters such as the handshapes. Each sign language has its inventory of occurring handshapes, which vary across sign languages (Stokoe, 1960; Friedman, 1975; Fenlon et al., 2015; Brentari et al., 2021). A small set of hand configurations has been identified as ‘unmarked,’ potentially occurring universally across sign languages (Klima and Bellugi, 1979; Boyes Braem, 1990; Marentette, 1995; Marentette and Mayberry, 2000; Sandler and Lillo-Martin, 2006; Henner et al., 2013; Caselli and Pyers, 2017). This identification is based partly on the role of these handshapes in two-handed signs (Battison, 1978).

There are several subcategories of two-handed signs. In *symmetrical* two-handed signs (e.g., ACCEPT and MOCK), both hands assume the same handshape, and there is no special restriction on the handshapes that can be used—they may be more or less complex. However, both hands must have the same location and movement (either simultaneous or in alternation) and the orientation must be symmetrical or identical. In contrast, *asymmetrical* signs (e.g., BUTTER and CONVINCEb) display restrictions on the handshape of the non-dominant hand (also known as the “weak” hand or H2). In an asymmetrical two-handed sign, the non-dominant hand is static (no independent movement) and limited to one of a small set of handshapes such as 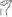, 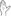, 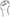, 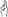 (Battison, 1978; Eccarius and Brentari, 2006). These configurations are considered unmarked (less complex) (Battison, 1978; Sandler and Lillo-Martin, 2006)^3^, while other hand configurations are considered marked (more complex).

Signs can also be phonologically more or less complex due to their syllable shape. Signed syllables can be defined by the types of movement used in a sign. Movement can consist of the hands moving from one location to another, describing a path movement. Path movement can be derived through changes in the position of the arm using the shoulder joint, and/or the elbow joint. Another kind of movement, known as local movement, involves hand position changes using the wrist joint, and/or changes in the hand configuration (e.g., closing from 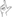 to 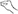; or opening from 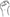 to 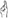), known as hand-internal movement. The vast majority of lexical signs are monosyllabic: they have at most one path movement (e.g., WEEK), or one local movement (e.g., MILKasym), or one path movement co-occurring with one local movement (e.g., THROW). More complex signs, with two (non-identical) sequential path movements (e.g., CENTER), or a path movement followed or preceded by a local movement (e.g., MAGIC), are rarely found in monomorphemic signs in ASL (Perlmutter, 1992; Brentari, 1998). More complex sequential movements that occupy more than one syllable are much less preferred than movement that occupies one syllable (Coulter, 1982; Liddell and Johnson, 1986; Sandler and Lillo-Martin, 2006).

In summary, phonological complexity for individual signs can be divided into two types: (a) simultaneous complexity (e.g., use of two hands or simultaneous movements); and (b) sequential complexity of disyllabic or multisyllabic signs (e.g., use of a sequence of non-identical movements). Given the affordances of the visual modality, simultaneous complexity may be more readily accommodated in sign languages than in spoken languages. Sign languages permit use of two hands, complex handshapes, and up to two types of movement in a single syllable, and they frequently combine morphemes into a single syllabic unit. On the other hand, sequential complexity is more common in spoken languages than in sign languages. Many spoken languages use words with complex sequential syllabic patterns not found in sign languages. We will return to discussion of these points in Section “Modality effects on complexity.”

### Development of sign phonology

**FIGURE 2:**
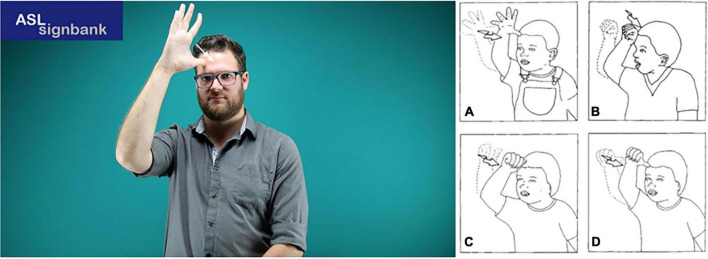
Handshape substitution errors produced by an ASL-acquiring child. **(Left)** Target form FATHERstr (reproduced with permission from ASL Signbank; Hochgesang et al., 2021). **(Right)** Child forms **(A–D)** [Copyright (2020) From Conlin et al. (2000: p. 60). Reproduced by permission of Taylor and Francis Group, LLC, a division of Informa plc].

As mentioned above, studies of the phonological acquisition of sign languages by children have primarily focused on describing signs using parameter-based analyses. Analysis of spontaneous production data from a variety of sign languages has revealed a consistent developmental pattern whereby location and (when included in analysis) orientation are controlled earlier than movement and handshape (Conlin et al., 2000; Morgan et al., 2007; Karnopp, 2008; Takkinen, 2008). Various factors potentially contribute to this hierarchy of relative parameter difficulty for signing children. For instance, the inventory of handshapes employed by sign languages is generally quite large compared to the inventory of locations. Contrastive handshapes are often distinguished by small differences in finger selection or position that young children do not yet possess the fine motor skills to manipulate. Conlin et al. (2000) note that in addition to a high error rate, handshape in early signing is also subject to a high degree of variability, sometimes even within a single filming session. Figure 2 shows four different handshape substitutions they illustrate for the target 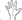 handshape of the ASL sign FATHERstr by a deaf child between 8–11 months of age.

Finally, some researchers have reported that young children are able to produce and recognize handshapes much earlier in isolation (e.g., as individual fingerspelled letters) than combined with a location and movement as part of a lexical sign (Siedlecki and Bonvillian, 1997), and even after they have mastered a given handshape in lexical signs, they may continue to make errors with that same handshape in the context of classifier constructions (Kantor, 1980).

In comparison with forming one’s hand into specific handshapes, moving the hands to a particular location of the body (e.g., the cheek versus the chest) demands much less precision and can thus be achieved by very young children (Siedlecki and Bonvillian, 1993). Of course, this does not mean that sign locations are uniformly target-like in early signing. Morgan et al. (2007) note that size of the target location affected accuracy for the British Sign Language (BSL)-acquiring subject they studied, who tended to replace relatively small target locations (e.g., the temple or the neck) with larger nearby locations (e.g., the cheek or the chest). Alternatively, location errors may be influenced by the saliency of the target location rather than its size, as suggested by Conlin et al. (2000) and Marentette and Mayberry (2000) for ASL, e.g., signing TELEPHONE at the ear rather than at the cheek. Under this account, some locations used by the child’s sign language are not yet included in their developing body schema (perhaps those for which the child does not yet have a label, e.g., “cheek” or “temple”) and thus are temporarily unavailable as locations for signs. Another characteristic location error pattern that has been reported by multiple researchers affects signs that require the hand to reach across the midline of the body. Bonvillian and Siedlecki (1996) and Conlin et al. (2000) report that for ASL signs such as BEAR, which requires both hands to cross and make contact with the opposite (contralateral) side of the torso, children avoid crossing the midline and instead contact the same (ipsilateral) side of the torso.

Movement accuracy in native signing children’s spontaneous production is often reported as falling somewhere between location and handshape accuracy (Siedlecki and Bonvillian, 1993; Conlin et al., 2000). Meier et al. (2008) attribute a large proportion of these movement errors to limitations in the child’s motor skills. For instance, they argue that the challenges of coordinating paired articulators are reflected in young children’s production of mirroring errors, in which signs that require the two hands to assume different handshapes and/or movements are instead produced with the same handshape and/or the same movement. Meier et al. (2008) also report that children appear to avoid two-handed signs with differing handshapes and/or movements (e.g., MEANING), noting that they occur with high frequency in adult ASL but are strikingly under-represented in deaf children’s spontaneous signing. Another movement error related to motor control is proximalization, or modification of the joints used to produce sign movement from those farther away from the torso (e.g., knuckles and wrist) to those more proximal to the body (e.g., elbow and shoulder). For instance, the ASL sign FATHERstr in Figure 2 (left) involves movement originating from the elbow joint. However, the child production illustrated in Figure 2A shows the movement from not only the elbow, but also the shoulder, a more proximal joint. Other instances of proximalized movement involve substitution of more proximal joints for less proximal joints, or in signs featuring multiple active joints, omission of more distal joints. These patterns are also attested in adult L2 signing and in child-directed signing (Holzrichter and Meier, 2000; Mirus et al., 2001).

Analyses of children’s spontaneous signing report that path movement is generally controlled earlier than hand-internal movement (Cheek et al., 2001) and signs that call for both path and internal movement at the same time are particularly challenging. Morgan et al. (2007) observe that the deaf child subject they studied (ages 19–24 months) modified the movement feature in roughly half of the signs she attempted, through (a) substituting a different path (circular movements were especially error-prone), (b) omitting, proximalizing, or substituting a sign’s internal movement, or simplifying signs that include both path and internal movements (mostly by deleting the path or internal movement, or by producing them sequentially rather than simultaneously).

The L1 sign language studies summarized here do not explicitly investigate the effect of sign complexity on acquisition, but we can deduce that some of the types of phonological complexity described in Section “Phonological complexity in sign languages” adversely affect the accuracy of children’s production of certain parameters and/or the overall sign. For instance, Meier et al. (2008) report that ASL-signing children of 8–17 months produced sympathy errors, which occur when the non-dominant hand unexpectedly copies the movement of the dominant hand. Such errors can be regarded as a reaction to the relative complexity of two-handed asymmetrical signs. Similarly, the observation that the same handshapes may be produced more accurately in isolation than in the context of a lexical sign or classifier construction suggests that the “added demands of simultaneously producing location and movement aspects may [make] the task of correct handshape formation too difficult” (Siedlecki and Bonvillian, 1997, p. 34). Finally, detailed error figures reported by Morgan et al. (2007) indicate an adverse effect of complexity on movement accuracy. They report that the child subject they studied (age 19–24 months) displayed errors in 100 of the 118 (85%) attempted BSL signs featuring simultaneous path and internal movement; this proportion of errors is much higher than for signs with either path or internal movement in which the path was incorrect (45% errors), or the internal movement was incorrect (46% errors).

### Studies of phonology using pseudoword/pseudosign tasks

A common method for assessing phonological processing skills in children is the use of non-word repetition tasks, also known as pseudoword tests. In English, two commonly used tasks are Children’s Test of Non-word Repetition (CNRep) (Gathercole and Baddeley, 1989) and the English Non-word Repetition Task (NRT) (Dollaghan and Campbell, 1998). These tests present children with novel words that are phonotactically permissible yet meaningless in their target language. Children hear and then reproduce the stimuli as accurately as possible, recalling the phonological form without relying on prior lexical knowledge. These tests for spoken language can be used to assess accuracy at the whole word level, and at the segmental level (consonants and vowels), as well as for various parameters at the suprasegmental level (stress, syllable, and tone).

Assessments using these tasks report a general trend for age of participants and length of pseudowords. Older children perform better than younger children, and shorter items are produced with higher accuracy than longer items (Chiat, 2006). This length effect has been found in English (Gathercole et al., 1994; Weismer et al., 2000; Thal et al., 2005), and other languages such as Brazilian Portuguese (Santos et al., 2006), Spanish (Ebert et al., 2008), Korean (Lee et al., 2013), Swedish (Sundström et al., 2014), French (dos Santos and Ferré, 2018), and Vietnamese (Pham et al., 2018). While some studies only include words of two or more syllables, others have found that when one-syllable words are included in the stimuli, such as the NRT, which consists of one- to four-syllable pseudowords in English, one-syllable and two-syllable words were produced with a similar accuracy level, with accuracy dropping only from three syllables upward by typically developing children (Gathercole et al., 1994; Weismer et al., 2000; Thal et al., 2005).

In the same vein as these spoken language tasks, sign-based non-word repetition tasks have also been developed following the same principles. Researchers have used pseudosign tasks to study the acquisition of British Sign Language (BSL) (Mann et al., 2010), Brazilian Sign Language (Libras) (Quadros et al., 2014), American Sign Language (ASL) (Cruz et al., 2014; Kozak, 2018), French Sign Language (LSF) (Cristini and Bogliotti, 2015); and with adults using Sign Language of the Netherlands (NGT) (Klomp, 2015; Vink, 2018). These tasks present phonotactically permissible but meaningless signs to participants, who then repeat them as accurately as possible. These tasks focus on the parameters of handshape, location, movement, and some include orientation as well.

In these tasks, it has generally been found that location is the most accurately reproduced parameter, and handshape is the least accurate. Furthermore, unmarked handshapes and simple movements (internal or path) are more accurately reproduced than marked handshapes and complex movements (which combine path movements with hand-internal movements and/or orientation change).

While these tasks are most commonly run unimodally, there have been studies comparing bimodal bilingual children’s phonological abilities on the non-word repetition tasks in both modalities; for American children as well as Brazilian children, finding a positive correlation for scores between spoken and signed modalities (i.e., English and ASL, or Brazilian Portuguese and Libras) (Cruz et al., 2014; Kozak, 2018).

## Materials and methods

### Materials

The ASL-based pseudosigns were developed following criteria described by Mann et al. (2010). Our task consisted of 39 nonsense signs that conform to the dominance and symmetry conditions of ASL (Battison, 1978) which constrain the possible forms between the two hands. The stimuli were developed by a group of deaf and hearing researchers, all native or fluent ASL signers (Quadros et al., 2015). The internal structures of the pseudosigns ranged from simple to complex in form, comprising eleven possible sign configuration categories, shown in Supplementary Table 1A. These pseudosigns were signed by a deaf native signer against a plain blue backdrop to create the video stimuli^4^. Test items were randomized and separated by a fade to black, during which participants were instructed to copy the pseudosign they had just seen.

For this study, we regrouped the 39 pseudosigns according to the following three variables: number of hands, simultaneous movement combinations, and movement sequence.

(i) Number of hands: the stimuli were classified into one-handed signs (*N* = 18) and two-handed signs (*N* = 21), which include symmetrical signs (*N* = 15) and asymmetrical signs (*N* = 6).
(ii) Movement combinations: the stimuli were classified according to the number of simultaneous movement types. Three categories were identified: (i) only one movement type, either (a) path movement or (b) handshape/orientation change (*N* = 20)^5^; (ii) two simultaneous movement types (path movement plus handshape change, or path movement plus orientation change) (*N* = 11); (iii) three simultaneous movement types (path movement, orientation change, and handshape change) (*N* = 8).
(iii) Movement sequence: the stimuli were grouped into (i) signs that involve a movement sequence, i.e., a combination of two successive path directions or path movement plus hand-internal movement (*N* = 3); and (ii) signs that contain no movement sequence, i.e., no successive path movements (*N* = 36). Note that items with repetitive path movement or oscillation^6^ were counted as occupying only one syllable or one movement in the phonology, even though temporally they contain multiple movements and are phonetically not short (Jantunen, 2015). They can co-occur with other types of movement in the same temporal span (Brentari, 1998; Jantunen and Takkinen, 2010; Sandler, 2017), so for this reason, they were counted in the “no movement sequence” category. Signs having no movement sequence are considered to occupy a single syllable, while signs having a movement sequence, i.e., more than one non-identical movement in sequence, correspond to two syllables in the prosodic structure (Brentari, 1998; Wilbur, 2011).

The combinatorial possibilities of the three complexity variables are twelve (i.e., 2*3*2), although only eight combinatorial options were included in our stimuli. For instance, we did not design any one-handed or two-handed pseudosigns that both involve three simultaneous movement types and contain a movement sequence. Even if actual signs with such phonological structures exist, they are rarely attested and are thus very marginalized in the ASL lexicon. Also, one-handed pseudosigns with two simultaneous movement types, and two-handed pseudosigns with only one movement type were not included, although such gaps did not affect the overall results and patterns we propose in this paper. The eight combinatorial possibilities covered by the stimuli are provided in Table 1.

**TABLE 1:** Combinatorial possibilities of complexity in the pseudosign stimuli.

Category	Complexity variables			Number of items (*N* = 39)
	Number of hands (one, two)	Number of simultaneous movement types (one, two, three)	Movement sequence (yes, no)	
1	One	One	No	*N* = 8
2	One	Two	No	*N* = 6
3	One	Three	No	*N* = 3
4	One	One	Yes	*N* = 1
5	Two	One	No	*N* = 11
6	Two	Two	No	*N* = 4
7	Two	Three	No	*N* = 4
8	Two	Two	Yes	*N* = 2

**FIGURE 3:**
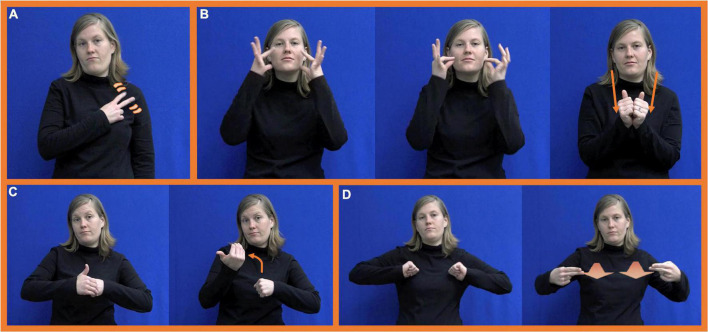
Still pictures of example pseudosign stimuli. **(A)** One hand, one movement type, no movement sequence (category 1). **(B)** Two hands, two simultaneous movement types, movement sequence (category 8). **(C)** Two hands, two movement types, no movement sequence (category 6). **(D)** Two hands, three simultaneous movement types, no movement sequence (category 7).

We provide illustrations of four pseudosigns as examples of our stimuli in Figure 3.

Apart from the three complexity variables above, a scale of handshape complexity was employed to examine possible associations between performance and handshape complexity. Handshape complexity was determined based on criteria developed by Eccarius and Brentari (2008) and Brentari et al. (2017), as described below.

Joint position and finger selection were assigned separate complexity scores. Joint position complexity scores of 1 (low) and 2 (medium) were given to shapes with fully open/closed fingers, and flexed fingers, respectively. The possible high complexity score of 3 was irrelevant to this study since our stimuli did not involve any joint positions like the 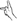 handshape or crossed fingers. Finger selection complexity scores of 1 (low) were assigned to selection of either all/no fingers or selection of index and/or thumb, 2 (medium) to pinkie finger or both index and middle fingers, and 3 (high) to other finger selections. A handshape was assigned an extra point each for involving change in joint position and change in finger selection, the former occurring in handshape contours and the latter in handshape contrast.

Brentari et al. (2017) did not discuss the complexity score of handshapes in two-handed signs. In our calculation of handshapes in two-handed pseudosigns, no extra points were assigned if the handshapes of the two hands were identical, but we added one extra point to two-handed pseudosigns in which the handshapes of the two hands were different.

Handshapes of various complexities were evenly distributed among the stimuli, so we do not consider classification of pseudosigns according to handshape complexity in Table 1. Further, handshape complexity was indexed as a continuous variable in this study whereas the other three variables were categorical variables, with each dividing the stimuli items into two or three groups in Table 1.

### Participants

Participants were 22 children (ages: 4;0–8;10, *$\bar{x}$* = 6;04, *SD* = 1;02) acquiring ASL as an L1 from Deaf parents^7^. Six were deaf (*$\bar{x}$* = 6;11, *SD* = 1;7), 3 deaf with cochlear implants (referred to as DDCI hereafter; *$\bar{x}$* = 5;07, *SD* = 0;1), and 13 hearing (referred to as kodas, or kids of deaf adults; *$\bar{x}$* = 6;03, *SD* = 1;0). Because all children were born into signing Deaf families, they were exposed to ASL from birth.

### Procedure

The test was run by native signers of ASL. Participants were told that they were going to see some silly signs and should try to copy them as well as they could. There were two unscored trial pseudosigns after the instructions, followed by the 39 target pseudosigns. Participants saw and reproduced the pseudosigns in a sitting position. All test items were shown only once, except in cases where a participant became distracted and missed an item.

### Scoring

The first author scored all 39 pseudosigns for accuracy at the feature level. During scoring, we encountered some sign reproductions that deviated from the target form in very subtle ways, and it was difficult to determine what degree of deviation counted as an error. Such challenges have also been reported by other researchers in scoring real signs reproduced by L2 signers (Willoughby et al., 2015; Ebling et al., 2021). To unify the scoring criteria and make a clear distinction between ‘errors’ and ‘distortions’ or acceptable deviations, we consulted two deaf researchers at Gallaudet University and discussed their intuitions on acceptable and unacceptable variations in thumb position, orientation and handshape of the non-dominant hand, height in neutral space, and oscillation. Having incorporated the deaf researchers’ judgments, the research team reached an agreement on the following scoring criteria at the feature level:

(1) Handshape: participants’ sign handshapes were coded for three aspects: finger selection, joint position, and thumb. The reproduction of each property was scored 1 if correct and 0 if incorrect. For compound-like pseudosigns that involve two contrastive handshapes, i.e., two sets of selected fingers, the initial and final handshapes were separately coded. In two-handed pseudosigns, handshapes on both hands were also separately coded. The handshape on the dominant hand was coded in the same way as handshape in one-handed pseudosigns. The handshape on the non-dominant hand was coded holistically, i.e., scoring 1 if it was reproduced correctly and 0 if any of the three properties, namely finger selection, joint position, or thumb, was reproduced inaccurately. One-handed pseudosigns scored 3 points or maximally 6 points (if there were two contrastive handshapes). Two-handed pseudosigns scored 3 points for the dominant hand and 1 point for the non-dominant hand. In sum, a system of 4 points or maximally 8 points (if there were two contrastive handshapes) was used for handshapes in two-handed pseudosigns.

**FIGURE 4:**
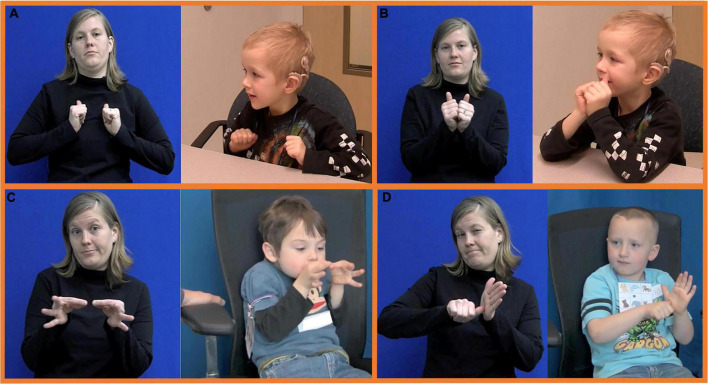
Target form and child form. **(A)** Thumb position; **(B)** height in neutral space; **(C)** height in neutral space; and **(D)** fingertips orientation of the non-dominant hand.

The following handshape errors were expected and identified: substitutions in finger selection, joint position, or thumb position; omission of handshape contrast (handshape contour that involves a change in selected fingers) which occurs when a reproduction involves a joint position change but fails to include a change of selected fingers; handshape assimilation of the non-dominant hand to the dominant hand. Reproductions of target handshapes with abducted fingers in which the fingers of the non-dominant hand were slightly splayed were not regarded as errors. Further, some slight deviations in the thumb position were not marked as errors. For instance, we coded as accurate instances where the thumb was slightly opposed (see Figure 4A, right), even though the thumb of the target form was fully unopposed, resting near the index finger (see Figure 4A, left). Other instances with more salient handshape deviations were marked as errors, such as the thumb being extended when it was closed/opposed in the target form.

(2) Location: participants’ sign locations were coded according to the height/side for each pseudosign; for those signs that inherently contain body/hand contact, the contact property was also coded, worth one extra point. The reproduction of each property was scored 1 if correct and 0 if incorrect. In compound pseudosigns that involve two major locations, both the initial and final locations were coded. The location on the dominant hand in two-handed pseudosigns was coded in the same way as in one-handed pseudosigns, but the location of the non-dominant hand was coded holistically. One-handed pseudosigns produced in neutral space were scored up to 1 point for location. One-handed pseudosigns with body contact were scored up to 2 points, and two-handed pseudosigns with body or hand contact were scored up to 3 points. Finally, in pseudosigns involving two major locations, the points were doubled in coding for a maximum of 6 points.

Location errors include substitution of the ipsilateral for contralateral side, omission of body/hand contact, and substitutions in height on the face or torso. We accepted some deviations in the height of signs in neutral space. For instance, some signs were produced in locations that were raised or lowered compared to the target as an accommodation of signing in a sitting position, or while leaning on the table (see Figure 4B, right). Some pseudosigns reproduced higher than the target in neutral space were accompanied by exaggerated non-manual signals (e.g., head forward and shoulders hunched up) to look “silly” (see Figure 4C, right)^8^. Because height in neutral space is not lexically contrastive in ASL (McBurney, 2002), we coded these instances of raised or lowered spaces as acceptable variants of the target.

(3) Movement: participants’ sign movements were coded for the following four properties: direction, repetition, shape, and alternation. Movement repetition was coded if the pseudosign inherently involved repetitive movement or oscillation, worth one extra point. If the movement path was anything other than straight, that movement trajectory/shape was coded, also worth one extra point. For pseudosigns that involve a movement sequence, the first and second movements were separately coded. Movement of the dominant hand in two-handed pseudosigns was coded in the same way as in one-handed pseudosigns. Movement of the non-dominant hand was coded holistically. In two-handed alternating pseudosigns, alternation was coded as an additional property of movement, adding one extra point. Finally, directions in path movement, handshape change, and orientation change were coded separately depending on the number of simultaneous movement types in the pseudosigns. Given that some two-handed pseudosigns contain as many as three simultaneous movement types, we did not collapse all movement properties into one point in the coding of the non-dominant hand, as we did for other parameters. 1 point was assigned to each type of movement (path movement, handshape change, or orientation change) on both hands if produced correctly, and 0 points were assigned if the property in question was produced incorrectly or completely lost.

Movement errors include omission or substitution of movement direction (handshape change, path movement, and orientation change), omission of repetition, and addition of unexpected movement. The deaf researchers we consulted were especially sensitive to differences in movement, particularly path movement, and hence a stricter standard on path movement was set. In contrast, the deaf researchers regarded children’s slower oscillation in response to signs featuring rapid alternations between fingers as completely acceptable. For targets with oscillating movement, we thus coded slowed repetitive finger movement as an acceptable variant, but failure to alternate fingers was still coded as a movement error.

(4) Orientation: participants’ sign orientation was coded for two properties: palm orientation and fingertip facing (i.e., orientation of the leading edge of the fingers). In one-handed pseudosigns, 1 point was awarded for orientation of the palm if it was reproduced correctly and 0 points if not. In a similar vein, 1 point was awarded for correct orientation of fingertips. In two-handed pseudosigns, accuracy was worth 3 points in total, with the dominant hand being assigned 2 points and the non-dominant hand 1 point^9^. If the pseudosigns involved an orientation change, both the initial and final orientations were scored independently.

Orientation errors were identified in substitution of the hand parts (radial, ulnar, palm, fingertips, back, and wrist) that contact certain body parts or the non-dominant hand. Deviations in orientation in neutral space were more acceptable to the deaf researchers we consulted and hence not regarded as errors unless the deviations are very salient. For instance, we observed many deviations in the fingertip orientation of the non-dominant hand. In one item, the fingertips point forward in the target, with the palm facing the side. Many children copied the item by positioning their non-dominant hand with fingertips pointing upward (see Figure 4D, right) rather than outward as in the target (see Figure 4D, left). The deaf researchers judged this subtle deviation as non-critical and arguably not erroneous as long as the palm was facing in the correct direction, i.e., to the side. But if the fingertips of the non-dominant hand pointed inward rather than outward, as observed for one child, this deviation was far more salient and was judged by our consultants as ‘awkward’ and not acceptable.

Overall accuracy for each pseudosign was calculated by dividing the total points earned for correctly reproduced features by the maximum possible number of points for that sign. Feature scores related to the same parameter were averaged to calculate composite accuracy scores for the individual parameters.

As a reliability check of our scoring system, the third author independently scored at the parameter level. We sampled 20% of the reported data (all 39 pseudosign reproductions from one deaf, one DDCI, and two koda participants). To render the scoring results between the two raters comparable, feature scores related to the same parameter were converted to binary scores of 0 and 1, with 0 indicating no parameter errors (i.e., 100% accuracy under the feature-based scoring approach) and 1 for parameter errors (i.e., <100% accuracy under the feature-based scoring approach). The inter-rater agreement for location, handshape, orientation, and movement was 90, 88, 87, and 86%, respectively.

## Results

The performance of each participant was measured by accuracy in the reproduction of pseudosigns. We examined both overall accuracy and individual parameter accuracy. Complexity was measured by four variables: number of hands (two-handed vs. one-handed), movement combinations (less than three simultaneous movement types vs. three simultaneous movement types), presence of movement sequence (movement sequence vs. no movement sequence), and handshape complexity. We will report results from both univariate analysis and multivariate logistic regression models.

### Overall accuracy

The overall accuracy on the pseudosign repetition task across all 22 children averaged 91.4% (*SD* = 11.2%) The average accuracy was 96.0% (*SD* = 7.3%) in the deaf group (*N* = 6), 91.4% (*SD* = 10.0%) in the DDCI group (*N* = 3) and 89.3% (*SD* = 12.2%) in the koda group (*N* = 13).

Regarding performance on signs with various degrees of complexity, we compared the accuracy score by number of hands, number of simultaneous movement types, and movement sequence, as shown in Table 2.

**TABLE 2:** Accuracy in signs that vary by number of hands and movement combinations (simultaneous and sequential).

Pseudosign complexity (number of hands * number of simultaneous movement types * number of movement sequence)	Avg. accuracy (*SD*), %
One-handed, one movement type, no movement sequence (*N* = 8)	92.9 (3.8)
Two-handed, two simultaneous movement types, no movement sequence (*N* = 4)	92.7 (5.7)
One-handed, two simultaneous movement types, no movement sequence (*N* = 6)	92.5 (6.1)
Two-handed, one movement type, no movement sequence (*N* = 11)	92.5 (6.0)
Two-handed, three simultaneous movement types, no movement sequence (*N* = 4)	88.9 (9.1)
One-handed, one movement type, movement sequence (*N* = 1)	87.7 (15.7)
One-handed, three simultaneous movement types, no movement sequence (*N* = 3)	86.6 (6.7)
Two-handed, two simultaneous movement types, movement sequence (*N* = 2)	86.3 (11.6)

In Table 2, it can be seen that the pseudosigns involving a movement sequence scored among the lowest for accuracy (86.3% for items with two hands, two simultaneous movement types, and 87.7% for the item with one hand, a single movement type) and showed the greatest variability in accuracy. Further, among the pseudosigns with no movement sequence, pseudosigns with three simultaneous movement types had lower accuracy scores (86.6% for one-handed items and 88.9% for two-handed items) than those with one or two movement types (accuracy above 92.5%). Finally, no clear difference in accuracy was seen between one-handed and two-handed pseudosigns.

**FIGURE 5:**
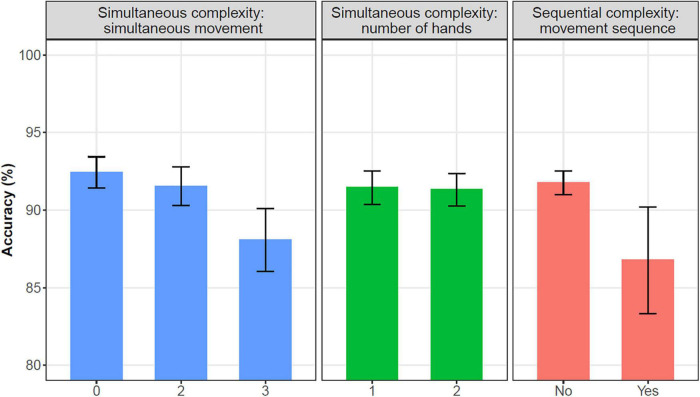
Overall accuracy (average across 22 participants) in items with differing simultaneous and sequential complexities, with 95% confidence intervals.

We divided complexity into two dimensions: simultaneous and sequential. Simultaneous complexity is displayed by number of hands (two-handed vs. one-handed) and number of simultaneous movement types (three, two or no simultaneous movement). Sequential complexity is manifested by the presence of a movement sequence (movement sequence vs. no movement sequence). In Figure 5, the average accuracy is compared across several complexity measures. The (non-)overlapping confidence intervals in Figure 5 indicate that some complexity measures were found to influence the overall accuracy, but some others were not. We found a significant difference in overall accuracy between signs with three simultaneous movement types and signs with one or two simultaneous movement types, although the overall accuracy in items with one movement type and two simultaneous movement types did not significantly differ. The items that involve a movement sequence had a significantly lower accuracy compared with items with no movement sequence. Finally, the overall accuracy difference between two-handed and one-handed items was not significant.

**FIGURE 6:**
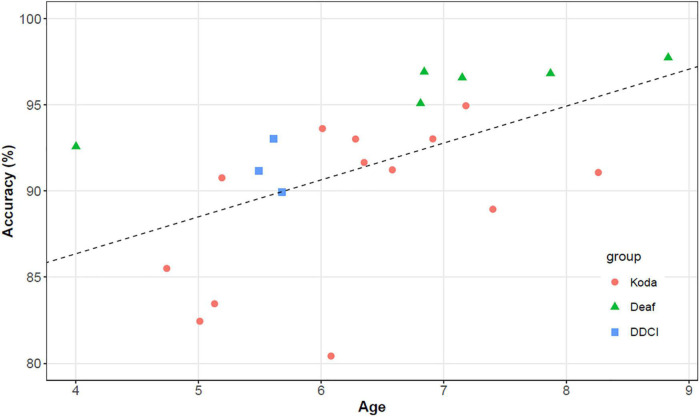
Overall accuracy and its relationships to age (intercept = 0.778, slope = 0.021, *p* < 0.001).

We also found a univariate association of age with the overall accuracy (intercept = 0.78, slope = 0.02, *p* < 0.001), as shown in Figure 6. This suggests that performance improved as age increased.

### Parameter accuracy

To obtain accuracy of each individual parameter, we averaged the feature scores related to the same parameter to calculate composite scores. In order to make our results comparable to other studies on sign phonological development, which predominantly used parameter-based scoring (see Section “Development of sign phonology”), we also calculated the parameter scores based on the less granular binary method. That is, we scored the production either as accurate (i.e., no errors in the production of this parameter) or as inaccurate (i.e., at least one error in the production of this parameter). The results of individual parameter accuracy, accuracy range, and number of errors from the two scoring methods are provided in Table 3.

**TABLE 3:** Accuracy, accuracy range, and number of errors distributed in each parameter.

Parameters	Feature-based scoring			Parameter-based scoring		
	Avg. accuracy (*SD*), %	Accuracy range, %	Number of errors	Avg. accuracy (*SD*), %	Accuracy range, %	Number of errors
Orientation	93.1 (3.5)	86.3 – 98.3	220	82.8 (8.2)	69.2 – 97.4	147
Location	92.1 (5.0)	81.1 – 99.4	228	79.3 (11.8)	56.4 – 97.4	177
Handshape	90.6 (6.0)	74.1 – 96.8	374	74.0 (12.6)	43.6 – 89.7	222
Movement	90.0 (6.8)	75.6 – 97.9	278	81.0 (11.2)	56.8 – 94.9	162

We predicted that the feature-based scoring method would yield relatively higher scores and lower variability than the parameter-based scoring method. The results in Table 3 show that this prediction was borne out. As introduced in Section “Scoring,” feature-based scoring provided more opportunities for participants to earn points for accurate reproduction of the various features under each parameter. Thus, accuracy is relatively high, and variability is low, indicated by the results of higher average accuracies and narrower accuracy range in feature-based scoring compared to parameter-based scoring.

In terms of number of errors, more errors occurred in handshape, followed by movement, location, and orientation in feature-based scoring. Further, feature-based analysis could capture multiple errors within each parameter, which were obscured under parameter-based scoring. Regarding accuracy score, movement was produced least accurately, followed by handshape, location, and orientation, based on feature-based scoring. Overall patterns were similar to the results of parameter-based scoring except that movement performance was better under parameter-based scoring, coming in as the second most accurate after the orientation parameter. The increased accuracy in movement could be ascribed to the fact that as shown in Table 3, the number of movement errors as well as handshape errors drastically decreased when switching from feature-based scoring to parameter-based scoring method. Combining accuracy scores and number of errors based on feature scoring, we found that location and orientation were produced more accurately than movement and handshape, in line with the literature on phonological accuracy in naturalistic production discussed in Section “Introduction.”

**FIGURE 7:**
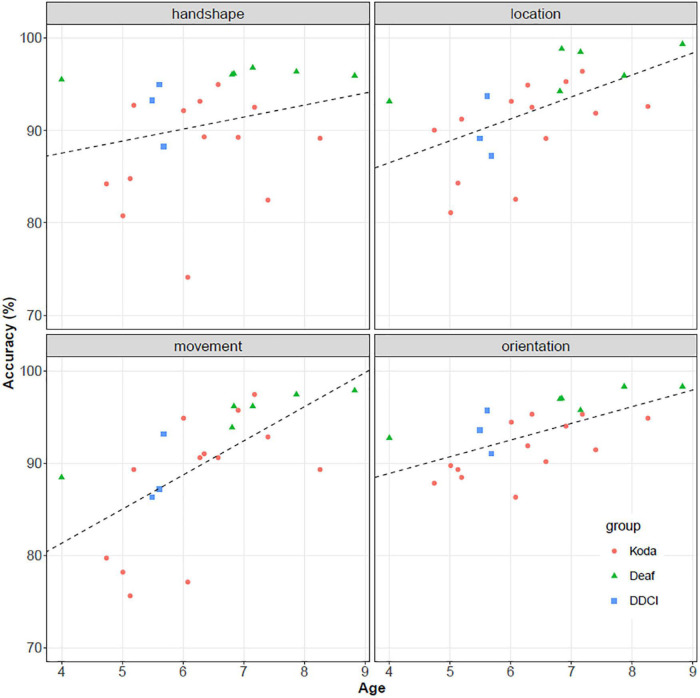
Individual parameter accuracies and their relationships to age.

We performed univariate regression models to analyze the association between individual parameter accuracy and age. After Bonferroni correction, increased age was found to be associated with better performance for almost all the parameters (location: intercept = 0.770, slope = 0.024, *p* < 0.01; orientation: intercept = 0.817, slope = 0.018, *p* < 0.01; movement: intercept = 0.666, slope = 0.037, *p* < 0.01) although the association between age and handshape accuracy was not statistically significant (intercept = 0.924, slope = 0.013, *p* = 0.24). These results of univariate association between parameter accuracy and age are provided in Figure 7. The lines are fitted by univariately regressing accuracy scores on age.

**FIGURE 8:**
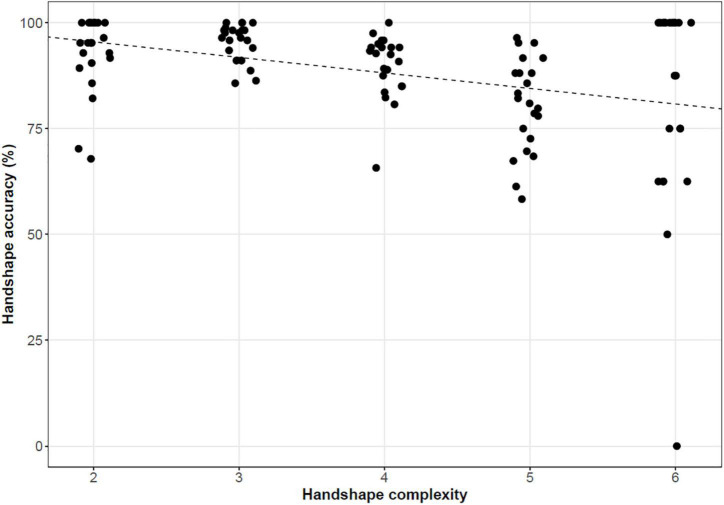
Handshape parameter accuracy and its relationships to handshape complexity (intercept = 1.029, slope = 0.027, *p* = 0.001).

In addition, as discussed in Section “Materials,” we examined the scoring of complexity for the handshape parameter (complexity score range: 2–6). A significant association was found between accuracy in handshape parameter and handshape complexity (*p* = 0.001). In Figure 8, the handshape complexity is slightly jittered to separate points and the line is fitted by univariately regressing handshape accuracy scores on handshape complexity. In addition, no significant association emerged between overall accuracy of the item and handshape complexity (*p* = 0.486).

### Multivariate analysis

Many of the factors we considered in this study can be correlated. For example, children in the deaf group are older than those in the other two groups, which could have given them an advantage on this task. Also, the different complexity measures of the items may also depend on each other. We thus conducted a multivariate analysis to jointly analyze the effects of each factor on the accuracy scores. We performed logistic regression with participant and item level random effects to analyze the association between accuracy scores and sign complexity, adjusting for age and group (koda, deaf, and DDCI). Phonological complexity for pseudosigns was measured by four variables: two-handed vs. one-handed (number of hands), three vs. two or no simultaneous movement type, movement sequence vs. no movement sequence, and handshape complexity. Logistic regression with binomial distribution was used to model the number of errors out of the total points in each pseudosign. The performance of a participant was measured by the errors of location, handshape, movement, and orientation. The model specification is provided in the Supplementary material shared on OSF. All the analyses were conducted using R software (R Core Team, 2021) and package lme4 (Bates et al., 2015).

Table 4 shows the result of the regression analysis. The odds ratios (OR, meaning the ratio of probability of making an error to the probability of not making an error), 95% confidence intervals (CI) and *p*-values are provided. An OR < 1 means the factor is associated with fewer errors and thus better overall performance. The following factors are significantly associated with better overall performance, with *p*-values less than 0.05: older age, being deaf, two or no simultaneous movement type, and no movement sequence. In contrast, the accuracy difference between two-handed and one-handed signs is not significant.

**TABLE 4:** Results of multivariate logistic regression of overall accuracy of the item on age, group, and complexity.

Factor	Reference	OR	95% CI	*P*-value
Intercept	n/a	0.365	(0.126, 1.053)	0.062
Age	1 year	0.761	(0.665, 0.871)	<0.001
Group: deaf	Koda	0.358	(0.248, 0.515)	<0.001
Group: DDCI	Koda	0.648	(0.419, 1.001)	0.051
Handshape complexity	increment of 1	1.083	(0.908, 1.292)	0.376
Complexity: 2-handed	1-handed	1.657	(0.662, 1.383)	0.815
Complexity: simultaneous movement, three	Two or no	2.208	(1.149, 4.24)	0.017
Complexity: movement sequence	No movement sequence	2.208	(1.149, 4.249)	0.017

Within the two-handed items, we compared the two subtypes, i.e., symmetrical signs and asymmetrical signs. No significant difference in overall accuracy (OR = 0.684, *p*-value = 0.210) was found between the production of these two subtypes. Details of multivariate analysis of accuracy in symmetrical signs and asymmetrical signs are provided in Supplementary Table 1B.

We also tested whether there is interaction between the complexity variables. Since some of the combination of complexity measures were not available in the pseudosign items, we were only able to test the interaction between handshape complexity and number of simultaneous movement types/movement sequence/hands, and between number of hands and number of movement sequence/simultaneous movement types. The interaction effects were not found to be statistically significant, as indicated in Supplementary Table 1C. That is, no significant interaction between two-handedness and three simultaneous movement types or movement sequence was found. No significant interaction was found between handshape complexity and the other three complexity measures. Based on the goodness of fit of the interaction model (i.e., the Akaike Information Criteria of the interaction model is greater than that of the main effect model), we conclude there is no interaction between complexity measures on the overall accuracy and therefore base our major findings on the main effect model reported in Table 4.

Regarding handshape, although handshape complexity is associated with handshape accuracy as reported in Section “Parameter accuracy,” no significant association was found between handshape complexity and overall accuracy of the item or performance on other parameters (movement, location, and orientation), as indicated in Table 5. With respect to effects of other parameters, Table 5 shows that the association between lower accuracy on orientation and having three simultaneous movement types is statistically significant (*p* = 0.014). The associations between the complexity measures (number of hands, number of simultaneous movement types, and number of movement sequence) and movement parameter accuracy are relatively strong, from which only movement sequence is statistically significant (*p* < 0.001). An elaborate version of Table 5 which contains the 95% confidence intervals and *p*-values is provided in Supplementary Table 1D.

**TABLE 5:** Association of each parameter accuracy with age, group, and item complexity measures.

Factor	Reference	Location OR	Handshape OR	Orientation OR	Movement OR
Intercept	n/a	1.309	0.032	0.249	1.327
Age	1 year	0.723***	0.864	0.745***	0.648***
Group: deaf	Koda	0.348***	0.268***	0.388***	0.386***
Group: DDCI	Koda	0.811	0.480	0.685	0.613
Complexity: handshape	Increment of 1	0.767	1.687***	1.003	1.015
Complexity: 2-handed	1-handed	0.773	1.349	1.468	0.625
Complexity: 3 SM	Two or no	1.870	0.944	3.047*	1.896
Complexity: mseq	No mseq	1.276	0.777	1.245	7.404***

3 SM, simultaneous movement types; mseq, movement sequence. *p < 0.05, ***p < 0.001.

## Discussion

This study investigated ASL-signing children’s phonological development using a pseudosign repetition task. It also examined possible relationships between accuracy in pseudosign repetition, age, and complexity in multiple dimensions. Through these investigations, we are able to comment on potential modality-based differences between phonological development in signed and spoken languages.

Previous studies examining performance on a variety of tasks have found slightly different patterns of accuracy across the four parameters. The ranking of parameters by observed accuracy can vary due to differences in task (perception/discrimination and production), stimuli complexity, and participants [children vs. adults; L1 signers vs. L2 signers vs. non-signers; as reported by Conlin et al. (2000), Mirus et al. (2001), Emmorey et al. (2009), and Mann et al. (2010)]. Here, we focus on comparing our results with general patterns reported in other studies of phonological development.

### Phonological development

In the current study, accuracy among the 22 ASL-signing children (mean age: 6;04) in the pseudosign repetition task was good, with an average of 91.4%. As indicated in Figure 5, overall, repetition accuracy increased with child age, echoing the results of other child pseudosign repetition tasks (Marshall et al., 2006; Mann et al., 2010; Cruz et al., 2014; Koulidobrova and Ivanova, 2020).

We also found that performance on individual parameters (handshape, location, movement, and orientation) increased with age, as shown in Figure 6, although the association between performance in handshape parameter and age was not significant. This somewhat surprising result can probably be explained by the fact that younger-aged children are already performing relatively well in handshape. A closer look at individual participants revealed that one koda participant underperformed with respect to their age. Given our relatively small sample size (22 children), a single outlier score could have a disproportionate effect on the distribution of the scores across all participants. We leave this for future research once a larger sample size can be guaranteed.

To examine signing children’s development patterns, we examined pseudosign accuracy for individual parameters. Our results revealed that movement and handshape were less accurately produced than location and orientation. These findings are consistent with accuracy patterns reported for real signs produced by both L1 (Conlin et al., 2000; Marentette and Mayberry, 2000; Morgan et al., 2007) and L2 signers (Jissink, 2005; Chen Pichler, 2011; Ortega and Morgan, 2015; Ebling et al., 2021). In the next subsections, we examine additional evidence from both child and adult learners that corroborate our finding of handshape and movement as the most error-prone parameters.

#### Handshape is difficult for everyone

As shown in Table 3 in Section “Parameter accuracy,” the accuracy of handshape is second lowest after movement, and handshape is more prone to errors as indicated by the fact that most feature-level errors were distributed in the handshape parameter. This pattern is consistent with diary studies of children acquiring ASL as a first language, in which handshape was controlled later than location and movement (McIntire, 1977; Boyes Braem, 1981; Siedlecki and Bonvillian, 1993). Studies of the development of ASL (Marentette and Mayberry, 2000; Cheek et al., 2001) and other sign languages (Clibbens and Harris, 1993; Takkinen, 2008; Lutzenberger, 2022) converge on the finding that handshape is produced with the most errors or modifications. Other pseudosign studies with signing children similarly report higher frequency of errors for handshape than for other parameters (Marshall et al., 2006; Mann et al., 2010; Cristini and Bogliotti, 2015).

There are several factors that potentially contribute to the disproportionately high error rate for handshape. One is the relatively large inventories of contrastive handshapes employed by sign languages, compared to smaller inventories of contrastive movements and locations (Meier et al., 2008; Orfanidou et al., 2009). With a greater number of distinct handshapes comes an increased level of detail that signers must attend to in order to distinguish between similar handshapes. Accordingly, the phonological representation for handshape is the most structurally complex, decomposable into smaller units of finger selection, joint position, and thumb position (Mandel, 1981; Brentari, 1998; van der Kooij, 2002; Sandler and Lillo-Martin, 2006), which correspond to the three properties of handshape we examined in this study. Accurate production of these handshape properties requires fine motor control of small, distal articulators (the fingers), demanding levels of coordination that often exceeds that of developing signers, whether young children (Conlin et al., 2000; Meier, 2005) or adult learners (Hohenberger et al., 2002; Emmorey et al., 2009; Ortega and Morgan, 2015; Mertz et al., 2022).

Within handshapes, our results also showed that those with higher complexity are reproduced with lower accuracy by ASL-signing children (age range: 4;0–8;10). This negative effect of handshape complexity has also been reported for pseudosigns reproduced by hearing adult native signers (referred to as codas, or children of deaf adults) of the Sign Language of the Netherlands/NGT (Klomp, 2015). In both cases, signers’ poorer performance on pseudosigns with phonologically complex handshapes is consistent with longitudinal studies that report later acquisition of more complex handshapes in signing children’s phonological development (Cheek et al., 2001; Karnopp, 2002; Morgan et al., 2007; Wong, 2008; Pan and Tang, 2017).

#### Movement is difficult for everyone

As summarized in Section “Development of sign phonology,” young signers display a variety of movement errors in their spontaneous production, many of which have been attributed to children’s incomplete motor development. However, this explanation may be too simplistic, given that many of these same movement error patterns are also observed among adult sign language learners. Noting frequent proximalization errors in the ASL of hearing adults learning a sign language as a second language (referred to as M2L2 or second modality second language learners) and to a lesser extent, even in deaf adult signers, Mirus et al. (2001) suggest that this type of error is a modality-specific pattern that arises when learners of any age are faced with the “new and complex motor skill” (Mirus et al., 2001, p. 14) of coordinating the hands and arms in ways prescribed by an L2 sign language. Similarly, Hilger et al. (2015) report highly variable spatiotemporal patterns in adult M2L2 signing that may require years of exposure and practice to stabilize.

Problems in perception and processing also contribute to movement errors for both children and adults. The simple fact that sign languages employ two types of movement, internal and path movement, that often occur simultaneously is in itself a source of difficulty for learner perception or processing (this point is discussed further in Section “Modality effects on complexity”). Rosen (2004) argues that errors in which adult ASL learners correctly produce the path movement but not the simultaneous internal movement (e.g., producing the ASL sign for INFORMATION with the correct forward path movement, but with simultaneous closing of the hands from the 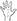 handshape to the 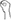 handshape rather than opening) reflect a perceptual error, and that these adult students possess the dexterity to produce the target form, but simply misremember the correct sequence of handshapes constituting the internal movement.

Other researchers have identified movement patterns that are particularly vulnerable to errors. Ebling et al. (2021) report especially high error rates for two movement patterns in their adult M2L2 learners’ reproduction of isolated Swiss German Sign Language (DSGS) signs. The first involves horizontal circular movements produced in the wrong direction, which can be considered a type of “mirror” error (Rosen, 2004). Such errors are common among M2L2 signers and are attributed to the signer failing to first rotate the sign to their own perspective; indeed, Quandt et al. (2021) recently documented poorer mental rotation ability for beginning signers compared to fluent signers. The second “specially marked movement” reported for DSGS are those involving a sequence of an outward path followed by a downward path. Ebling et al. (2021) note that this sort of movement sequence is relatively rare in DSGS, and viewed from straight on, is apparently misperceived (and subsequently misproduced) by inexperienced signers as a single downward arc movement. This analysis is consistent with reports from Bochner et al. (2011) that adult hearing M2L2 learners’ ability to discriminate movement contrasts in ASL is weaker than for other parameters, a finding replicated by Schlehofer and Tyler (2016) for other M2L2 learners of ASL. Similarly, Williams et al. (2016) report that adult learners misperceive sign movement more often than other parameters when viewing signed sentences.

Interestingly, although the movement parameter is most often misperceived (and thus misproduced) by learners, it is highly salient for experienced signers (Hildebrandt and Corina, 2002; Orfanidou et al., 2009). Examining M2L2 signers’ non-target reproductions of DSGS signs that were rated as severe errors by experienced deaf judges, Ebling et al. (2021) report that non-target movements account for the majority of these errors (61%), far outstripping the second most salient parameter, handshape (20%). In other words, not only is movement the most commonly misperceived and misproduced parameter, deviations in this domain also contribute the most to viewers’ perception of inaccurate or incorrect signing, suggesting that movement warrants additional attention in sign language pedagogy.

### Group differences

As indicated in Table 4, the deaf group and the DDCI group achieved higher accuracy scores than the koda group. It is noteworthy that performance on the pseudosign repetition task is positively associated with age (Marshall et al., 2006; Mann et al., 2010). As noted earlier, the children in the deaf group were older than those in the other groups, which could have given them an advantage on this task. Adjusted for age, the deaf group still outperformed the koda group, although no significant difference was found between the DDCI group and the other two groups. However, these results should be considered with caution since the sample size for these groups is small. Very little literature has compared deaf children, koda children, and DDCI children in any aspect of sign language development (but see Cruz et al., 2014; Reynolds, 2016; Kozak, 2018). In addition, there is a similar result comparing deaf and coda adults reported in Klomp (2018); she found that deaf adults were more accurate than codas in a NGT pseudosign repetition task.

We do not intend to make a claim here regarding group differences due to our small sample sizes and potential variations among the individuals’ ASL and English input, even though they were all exposed to ASL from birth. Kodas are heritage signers (Chen Pichler et al., 2018; Reynolds, 2018) and as observed in other studies of heritage language learners, it is reasonable to infer that hearing bimodal bilinguals may follow distinct developmental patterns for some aspects of their grammar compared to deaf native signers. We also note that considerable deviations in thumb position were identified in the koda group as compared to the deaf and DDCI children (Kozak et al., 2022), although such deviations were perceived as lying somewhere between acceptable variation and real errors, as evaluated by the deaf researchers we consulted. Thumb position deviations occurred often when the target form involves a 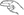 handshape but the participants, particularly children in the koda group, produced these items with their thumb extended. This 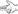 handshape is arguably a permissible variation in ASL real signs (Battison et al., 1975; Lucas, 2001) and is frequently attested in connected ASL production (Cheek et al., 2001) as well as in other sign languages (e.g., Ormel et al., 2017). This kind of more frequent occurrences of thumb deviations among kodas might be affected by age since the average age of koda group is younger than the deaf group. The younger-aged children may be less sensitive to the formality of the tasks and more easily bored by the pseudosign task, giving rise to more informal use of the thumb. The older-aged children, in contrast, may have been more successful at staying focused and inhibiting acceptable variations, resulting in more accurate psuedosign reproduction. We leave these postulations to be examined in future research with larger samples.

### Modality effects on complexity

When considering how complexity of pseudowords (signed or spoken) relates to accuracy in reproduction, it is important to consider potential effects of modality on phonological complexity. Since pseudoword tasks require participants to perceive and remember novel stimuli that are not part of their mental lexicon, cognitive skills in perception, memory, and production are all relevant.

Let us consider the working memory factor, which is heavily taxed in pseudoword tasks. For children acquiring spoken languages, serial working memory develops throughout childhood, permitting the rote recall of increasingly longer sequences, including non-words composed of longer sequences of syllables (Gathercole and Baddeley, 1989; Gathercole et al., 1994). Adult deaf signers typically score lower than hearing speakers in tasks that require temporal sequencing of linguistic chunks (e.g., Hall and Bavelier, 2011). On the other hand, some studies report superior spatial coding abilities in deaf signers (e.g., Wilson and Emmorey, 2003; see Giezen, 2021 for an overview). ASL (and other sign languages) often packages information into simultaneously produced multimorphemic monosyllabic forms, rather than making extensive use of temporal sequencing as do spoken languages. It is hypothesized that this difference is related to the exact components of the working memory system that are constrained by the sensory modalities, as summarized in these references (Wilson et al., 1997; Emmorey et al., 2017; Brentari, 2019).

This difference in working memory preferences for spoken languages (sequential units) versus signed languages (simultaneous units) leads us to consider predicted differences between observed performance on spoken and signed pseudoword repetition tasks. For signers, longer sequences of units belonging to a single pseudosign could cause greater memory demands, especially in comparison to unit sequences of the same length in spoken languages. For speakers, length differences should be observed but only for more complex sequences. On the other hand, signing children may well be able to handle simultaneous complexity of various sorts, with complexity effects seen only beyond a certain threshold.

The results of our study are consistent with these predicted differences. Remarkably, neither the presence of two hands nor the presence of two simultaneous movement types led to reduced accuracy. Only when three simultaneous movements were presented was accuracy affected. This indicates that greater simultaneous sign complexity is within the processing capacity of our child participants. On the other hand, signed stimuli that exhibited sequential complexity on a par with disyllabic spoken words were reproduced with reduced accuracy. This is in contrast to spoken pseudoword tasks, in which children generally begin to show a breakdown in accuracy only once the word length reaches three or more syllables (Thal et al., 2005; Gathercole, 2006; Pham et al., 2018, a.o). In the following subsections we discuss each of these results in turn.

#### Number of hands

On the purely motoric level, the use of two hands can be considered more complex than the use of one hand, as discussed in Section “Development of sign phonology” for deaf L1 sign learners (Meier et al., 2008) and hearing L2 sign learners (Ortega and Morgan, 2015). Learning to coordinate the handshapes and movement of the two hands, as well as the timing when each is available for production, requires time.

On the linguistic level, two-handed signs are not simply equivalent to two one-handed signs. In fact, the non-dominant hand (sometimes called the “weak” hand or H2) is very limited in what it can do within two-handed lexical items. Battison (1978) classified signs depending on number of hands and suggested constraints on the non-dominant hand, as summarized in Section “Sign language phonology” above. Battison’s proposed symmetry and dominance conditions severely restrict the ways that the non-dominant hand is used. Other phonological models since then (Sandler, 1993; van der Hulst, 1996; Brentari, 1998; van der Kooij, 2002) have also emphasized the dependent status of the non-dominant hand. In addition, we did not find a difference in the overall accuracy of the two subtypes of two-handed items, namely symmetrical and asymmetrical signs. This suggests that regardless of how the non-dominant hand is restricted in a two-handed item (i.e., whether being subject to the symmetry condition or dominance condition), the presence of two hands does not impose difficulties on our child participants in this study.

These observations, together with our conception of simultaneous vs. sequential complexity, provide a reasonable explanation for the lack of an accuracy effect for pseudosign stimuli requiring the use of two hands vs. one hand.

#### Simultaneous movement combinations

As described in Section “Materials” above, the stimuli in our study were classified according to the number of simultaneous movement types involved: one (either path or handshape/orientation change), two, or three simultaneous movement types. Our results showed a significant effect on accuracy for signs with three movement types compared to those with one or two: specifically, overall accuracy is significantly lower for the signs with three co-occurring movement types. When faced with three simultaneous movement types, the participants in our study tended to eliminate the orientation change, maintaining path movement and handshape change.

This response pattern may be related to a proposed phonotactic constraint within an ASL syllable whereby either handshape or orientation may change, but not both (Wilbur, 1993; Uyechi, 1995; Brentari, 1998; Sandler and Lillo-Martin, 2006)^10^. Studies in spoken languages report that pseudowords are more likely to be reproduced correctly if their structure is consistent with real words (Gathercole et al., 1991; Chiat, 2006), and pseudosigns with three simultaneous movement types violate this phonotactic constraint in ASL. Along a similar vein, Orfanidou et al. (2010) observe that deaf signers are quicker to identify real BSL signs embedded between pseudosigns if the pseudosigns are wordlike, i.e., if they resemble real BSL signs. It is very likely that children in our study perceived items with three simultaneous movement types as less wordlike than other forms with fewer movement combinations, negatively impacting their accuracy.

We conclude that the complexity associated with two simultaneous movement types is within the processing capacity of our participants. This also reflects reports that signs with one or two movement types far outnumber signs with three simultaneous movement types (Brentari, 1998 for ASL; Jantunen and Takkinen, 2010 for Finnish Sign Language). At first glance, our finding appears to contrast with the longitudinal results of Morgan et al. (2007) summarized in Section “Development of sign phonology,” as well as a BSL pseudosign study conducted by Mann et al. (2010). The longitudinal study reported high error rates for real BSL signs with two simultaneous movement types, but the child in that study was much younger (19–24 months) than the children in the current study, making it likely that she was at a much earlier stage of motor and phonological development. The children studied by Mann et al. (2010) ranged from 3 to 11 years old and were observed to frequently simplify signs with two simultaneous movements [i.e., a simultaneous path movement and handshape change, referred to as “movement clusters”’ by Mann et al. (2010)] through deletion of one of the movements. However, this error pattern occurred mostly in pseudosigns that also contained a complex handshape, reflecting an effect of combined handshape and movement complexity rather than of the movement cluster itself. As reported in Section “Multivariate analysis,” we did not find a significant effect of handshape complexity on the performance in movement parameter, simultaneous movement types, although a significant effect was found on performance in movement sequence. Since Mann et al. (2010) did not test pseudosigns with more than two simultaneous movement types, the two studies are not directly comparable on this point.

#### Sequential movement combinations

The results of our pseudosign repetition task show that disyllabic forms, i.e., those with a movement sequence, were produced less accurately than monosyllabic forms, i.e., those without a movement sequence. This implies that processing capacity for signs at the sequential level is highly limited, a manifestation of modality effects related to the dominance of simultaneous structuring in visual languages, as discussed earlier.

Pseudoword repetition tasks in spoken languages have generally found effects for length of the stimulus (in syllables). While such tasks are frequently used with school-age children and are often employed for diagnosing developmental language disorder or delays (e.g., Dollaghan and Campbell, 1998), they have also been used with children as young as 2–4 years of age (Roy and Chiat, 2004). The length effects show that typically-developing English-speaking children (age range: 4;0–8;11) usually reproduce one- and two-syllable words more accurately than longer words (Dollaghan and Campbell, 1998; Weismer et al., 2000; Thal et al., 2005). This can be taken as an indication that disyllabic words are not particularly demanding, but longer sequences do require greater phonological working memory for spoken words. Although not all pseudosign studies include monosyllabic stimuli, it can be concluded that children around the same age as those in our study are highly successful with at least disyllabic words. In spoken stimuli with more than two syllables, a length effect is generally found, such that the greater the number of syllables, the more errors there are in reproduction. These results indicate that two-syllable spoken pseudowords have a different phonological complexity status than two-syllable pseudosigns.

The preference for monosyllables in pseudosigns reflects the common pattern of real signs consisting of a single syllable. Disyllabic signs exist, but they are rare, a distribution pattern that could be considered as reflecting a strong preference for signs to have at least one movement, but not more than one movement sequence in the temporal dimension (Coulter, 1982; Sandler, 1989; Wilbur, 1993; Brentari, 1998; van der Hulst and van der Kooij, 2021). Furthermore, when multisyllabic signs are found (e.g., in sign compounds), they often undergo phonological processes that reduce them toward the shape of a monosyllable (Liddell and Johnson, 1986; Brentari, 1998). Those findings are consistent with the results from our study, which indicate that children are able to apply such processes to reduce complexity, rendering pseudosigns more like the canonical form of real signs. The fact that sequential pseudosigns showed a lower reproduction accuracy tells us that again, wordlikeness is at play, since items with a movement sequence do not resemble the canonical form of real signs.

## Limitations and future directions

The results of this study indicate that complexity affects phonological development for signing children, but that they are not equally sensitive to all types of complexity. Specifically, signs with sequential movement complexity negatively impacted accuracy, while those with some simultaneous movement complexity did not. This finding raises intriguing questions about modality and language development to be addressed in future research. For instance, closer examination of the effects of simultaneous complexity on spoken language development would clarify whether the differential effects of simultaneous vs. sequential complexity we observed for signers also occurs for spoken language learners, e.g., perhaps in the context of complex tone patterns (Pham et al., 2018) or accent patterns (Sundström et al., 2014) accompanying speech. Additionally, expanding our investigation to other populations of sign language learners is important for understanding the effect of additional factors such as age of exposure (AoA) and quality of early sign language input. Our study examined only participants who had the benefit of exposure to a natural sign language from birth, a privilege limited to only a very small percentage of deaf or hard of hearing (DHH) children worldwide [fewer than 3.9% in the United States, according to Mitchell and Karchmer (2004)]. A much larger percentage of DHH children experience limited or delayed first language acquisition, a factor that has been shown to impact patterns in phonological accuracy on real signs among deaf adult signers (Nielson and Mayberry, 2021). Other pseudosign repetition studies have investigated signing children from Deaf families compared to those from hearing families (Quadros et al., 2012; Cristini and Bogliotti, 2015) and found that those from Deaf families achieved higher scores on the task compared to those from hearing families.

Additionally, our task was limited in scope, including only 22 participants across three groups whose ages were not balanced. Mann et al. (2010) investigated pseudosign repetition among BSL signers across a wider age range than ours and found a correlation between age and accuracy. Expanding our current cohort in both number and ages will be necessary for a more comprehensive investigation of the nature of group effects throughout childhood. Adult native signing controls should also be introduced for comparison purposes to the experimental groups.

Finally, while other studies involving these same participants have investigated pseudosign reproduction accuracy and its correlation to other phonological abilities (Cruz et al., 2014; Kozak, 2018), further investigation with wider scope should be conducted to see how these skills overall interact with participants’ phonological recall ability.

## Conclusion

In this study of pseudosign repetition task with L1 ASL-signing children, we found that children’s overall accuracy increased by age, and the accuracy on individual sign parameters was relatively high for location and orientation, but lower for handshape and movement. We reported that items with no movement sequence are significantly associated with better performance. Also, children achieved significantly better performance on items with a single movement or two simultaneous movement types than those with three simultaneous movement types. Finally, accuracy scores between two-handed and one-handed items were not significantly different. We conclude that simultaneous versus sequential phonological structure differ as sources of complexity in signed and spoken (non-)words. This modality effect in turn influences children’s processing patterns in the signed modality. The ability of signing children to process multiple simultaneous components, as revealed in our study, informs their phonological development in the visual-gestural modality. In light of these findings, we advance the employment of phonological complexity in the assessment of working memory and phonological skills in psycholinguistic studies of both spoken and signed languages.

## Data availability statement

The datasets presented in this study can be found at: https://osf.io/93cne/?view_only=0a7e72095d824a7db3b08453e6dc3e34.

## Ethics statement

The studies involving human participants were reviewed and approved by the Institutional Review Boards of the University of Connecticut and Gallaudet University. Written informed consent to participate in this study was provided by the participants or their legal guardian/next of kin. Written informed consent was obtained from the individual(s), and minor(s)’ legal guardian/next of kin, for the publication of any potentially identifiable images or data included in this article.

## Author contributions

SG conducted the primary analyses and wrote a first draft of multiple sections. LK conducted additional analyses. DL-M and DC supervised the project and acquired the funding. All authors contributed to conception, drafting and revising the manuscript, and approved the submitted version.

## Footnotes

1 Throughout this manuscript we only attend to manual parameters and generally do not discuss non-manual markings. The term ‘parameter’ should be understood as referring only to manual components here.

2 In sign language research, individual signs are typically named by using a gloss in upper-case which is a close translation equivalent for at least one sense of the sign. We adopt the identification glosses used in the ASL Signbank (aslsignbank.haskins.yale.edu; Hochgesang et al., 2021), which sometimes employ additional symbols (such as the bb on KNOWbb) to demarcate the specific sign intended. Readers can view videos of the signs glossed in this paper at that website through the links provided in the Supplementary material.

3 The dominance condition just described is one criterion for identifying unmarked handshapes in ASL. Other diagnostics of unmarked properties include but are not limited to order of acquisition, accuracy in repetition, complexity in the phonological structure, frequency of occurrence in the lexicon (van der Kooij, 2002; Sandler and Lillo-Martin, 2006).

4 The ASL pseudosign stimuli videos can be found at this link: https://slla.lab.uconn.edu/wp-content/uploads/sites/1793/2019/02/ASL-pseudosign-stimuli-random-SLLA.mov.

5 In this study, handshape change contributes to movement complexity as in the Prosodic Model (Brentari, 1998, 2019). Meanwhile, as discussed at the end of Section “Materials,” following Brentari et al. (2017), in the calculation of the complexity of the handshape parameter *per se*, we included the dynamic aspects so that an extra point is added if there is a change in joint position, and another extra point if there is a change in selected fingers.

6 According to Brentari (1998), repetition refers to a movement that is repeated; oscillation (also called trilled movement) refers to an uncountably repeated movement.

7 All the deaf children in our current study were raised in the Deaf community and are users of a natural sign language (ASL). Previous publications (including many of our own) used the capitalized term “Deaf” to designate individuals who identify as culturally Deaf, and who by extension use sign language as a principal mode of communication. However, this convention is changing, and we recognize the problematic nature of assuming the cultural identity of participants in our research, including young children who may not have adopted the Deaf identity label for themselves. We thus refer to “deaf children” and “deaf researchers” in this paper, although we retain the terms “Deaf community” or “Deaf families”.

8 This example of a ‘silly-looking’ pseudosign production comes from a child whose data were not included in the currently analysis, but for whom we have permission to publish images.

9 One reviewer suggested investigating accuracy in the symmetrical orientation and identical orientation between the two hands in two-handed signs. Participants in this study rarely made errors on orientation between the two hands. Further, the authors found that this distinction between symmetrical orientation and identical orientation in two-handed signs does not play a role in the contrast this study is focusing on, namely simultaneous versus sequential complexity.

10 One reviewer pointed out that the wrist is rarely contrastive, and wrist movement is most often an enhancing effect of hand opening/closing and therefore more phonetic than phonological in ASL real signs (Brentari, 2019). The authors carefully examined the stimuli and concluded that these enhanced orientation changes as a secondary effect of hand-internal movement were not found in the pseudosign stimuli that were tested in this study. The orientation change in the stimuli design is not a phonetic effect, but contrastive.
