# Supplementary material for: Phonological development in American Sign Language-signing children: Insights from pseudosign repetition tasks
Source: Front Psychol. 2022 Sep 8;13:921047. doi: 10.3389/fpsyg.2022.921047 (PMC9496651; doi:10.3389/fpsyg.2022.921047)
Supplement: Supplementary file 1 [file Data_Sheet_1.pdf]

Phonological development in American Sign Language-signing children: Insights from pseudosign repetition tasks

Shengyun Gu[1*], Deborah Chen Pichler[2], L. Viola Kozak[2], Diane Lillo-Martin[1]

[1]Department of Linguistics, University of Connecticut, Storrs, CT, USA

[2]Department of Linguistics, School of Languages, Education and Cultures, Gallaudet University, Washington, D.C., USA

*** Correspondence:**
Shengyun Gu
shengyun.gu@uconnn.edu

DOI 10.3389/fpsyg.2022.921047

Supplementary material – Pseudosigns

Web addresses for viewing videos of signs cited in the manuscript. Viewers can also go to https://aslsignbank.haskins.yale.edu/ and type the gloss in the 'Search gloss' box.

Glosses are listed in the order in which they appear in the text.

KNOWbb	https://aslsignbank.haskins.yale.edu/dictionary/gloss/554/
THINK	https://aslsignbank.haskins.yale.edu/dictionary/gloss/398/
DISAPPOINT	https://aslsignbank.haskins.yale.edu/dictionary/gloss/1258/
ALL-DAY	https://aslsignbank.haskins.yale.edu/dictionary/gloss/7/
ANNOTATE	https://aslsignbank.haskins.yale.edu/dictionary/gloss/3061/
ACCEPT	https://aslsignbank.haskins.yale.edu/dictionary/gloss/2045/
MOCK	https://aslsignbank.haskins.yale.edu/dictionary/gloss/3006/
BUTTER	https://aslsignbank.haskins.yale.edu/dictionary/gloss/2144/
CONVINCEb	https://aslsignbank.haskins.yale.edu/dictionary/gloss/1138/
WEEK	https://aslsignbank.haskins.yale.edu/dictionary/gloss/1418/
MILKasym	https://aslsignbank.haskins.yale.edu/dictionary/gloss/506/
THROW	https://aslsignbank.haskins.yale.edu/dictionary/gloss/1222/
CENTER	https://aslsignbank.haskins.yale.edu/dictionary/gloss/653/
MAGIC	https://aslsignbank.haskins.yale.edu/dictionary/gloss/666/
FATHERstr	https://aslsignbank.haskins.yale.edu/dictionary/gloss/764/
TELEPHONE	https://aslsignbank.haskins.yale.edu/dictionary/gloss/546/
BEAR	https://aslsignbank.haskins.yale.edu/dictionary/gloss/394/
MEANING	https://aslsignbank.haskins.yale.edu/dictionary/gloss/186/
INFORMATION	https://aslsignbank.haskins.yale.edu/dictionary/gloss/3945/

Table 1A | Distribution of pseudosign configurations

**Category**	**Number of pseudosigns**	**Pseudosign configurations**
**a**	N=3	One hand, one handshape, no path movement (hand-internal movement)
**b**	N=3	One hand, one handshape, path movement
**c**	N=3	One hand, handshape change, no path movement
**d**	N=3	One hand, handshape change, path movement
**e**	N=3	Two hands, one handshape, symmetrical movement
**f**	N=3	Two hands, one handshape, alternating movement
**g**	N=3	Two hands, handshape change, symmetrical movement
**h**	N=3	Two hands, two handshapes (H1 and H2 differ), no path movement
**i**	N=3	Two hands, two handshapes (H2 and H2 differ), handshape change on H1
**j**	N=6	Compound; one hand, handshape change, location change (major body area)
**k**	N=6	Compound; two hands, handshape change, location change (major body area)

Table 1B | Results of multivariate logistic regression of item accuracy on age, group, and two-handed item type (symmetrical vs. asymmetrical)

Factor	Reference	OR	95% CI	p-val
**Intercept**	n/a	0.634	(0.207,1.941)	0.424
**Age**	1 year	0.762	(0.642,0.903)	0.002
**Group: deaf**	koda	0.271	(0.171,0.430)	<0.001
**Group: DDCI**	koda	0.624	(0.364,1.070)	0.086
**Asymmetrical**	symmetrical	0.684	(0.378,1.238)	0.210

Table 1C | Results of multivariate logistic regression of item accuracy on age, group, and item complexity, including the interactions of complexity measures (SM: simultaneous movement)

Factor	Reference	OR	95% CI	p-val
**Intercept**	n/a	0.291	(0.079, 1.071)	0.063
**Age**	1 year	0.761	(0.665, 0.871)	<0.001
**Group: deaf**	koda	0.358	(0.248, 0.516)	<0.001
**Group: DDCI**	koda	0.649	(0.419, 1.002)	0.051
**Complexity: handshape**	increment of 1	1.153	(0.862, 1.54)	0.506
**Complexity: 2-handed**	1-handed	2.529	(0.648, 9.877)	0.182
**Complexity: simultaneous movement, three**	two or no	0.267	(0.034, 2.077)	0.208
**Complexity: movement sequence**	no movement sequence	1.754	(0.418, 7.365)	0.443
**2-handed*3 SM**	1-handed, two or no SM	0.792	(0.321, 1.955)	0.613
**2-handed * movement sequence**	1-handed, no movement sequence	0.708	(0.151, 3.315)	0.661
**Handshape * 2-handed**	handshape, 1-handed	0.769	(0.523, 1.131)	0.182
**Handshape *3 SM**	handshape, two or no SM	1.624	(0.987, 2.672)	0.057
**Handshape * movement sequence**	handshape, no movement sequence	1.175	(0.754, 1.829)	0.478

Table 1D | Association of each parameter error with age, group, and item complexity measures (3 SM = 3 simultaneous movement types, mseq = movement sequence)

Error type	Factor	Reference	OR	95% CI	p-value
**Location**	Intercept	n/a	1.309	(0.228, 7.518)	0.763
	Age	1 year	0.723	(0.593, 0.882)	0.001
	Group: deaf	koda	0.348	(0.199, 0.609)	<0.001
	Group: DDCI	koda	0.811	(0.446, 1.474)	0.491
	Complexity: handshape	increment of 1	0.767	(0.537, 1.096)	0.125
	Complexity: 2-handed	1-handed	0.773	(0.370, 1.611)	0.492
	Complexity: 3 SM	two or no	1.870	(0.723, 4.838)	0.196
	Complexity: mseq	no mseq	1.276	(0.339, 4.802)	0.718
**Handshape**	Intercept	n/a	0.032	(0.006, 0.164)	<0.001
	Age	1 year	0.864	(0.713, 1.047)	0.135
	Group: deaf	koda	0.268	(0.157, 0.457)	<0.001
	Group: DDCI	koda	0.480	(0.256, 0.898)	0.022
	Complexity: handshape	increment of 1	1.687	(1.243, 2.290)	0.001
	Complexity: 2-handed	1-handed	1.349	(0.712, 2.555)	0.358
	Complexity: 3 SM	two or no	0.944	(0.418, 2.133)	0.89
	Complexity: mseq	no mseq	0.777	(0.229, 2.636)	0.686
**Orientation**	Intercept	n/a	0.249	(0.053, 1.177)	0.079

	Age	1 year	0.745	(0.643, 0.863)	<0.001
	Group: deaf	koda	0.388	(0.250, 0.599)	<0.001
	Group: DDCI	koda	0.685	(0.440, 1.066)	0.093
	Complexity: handshape	increment of 1	1.003	(0.710, 1.416)	0.985
	Complexity: 2-handed	1-handed	1.468	(0.707, 3.050)	0.303
	Complexity: 3 SM	two or no	3.047	(1.251, 7.417)	0.014
	Complexity: mseq	no mseq	1.245	(0.350, 4.433)	0.735
**Movement**	Intercept	n/a	1.327	(0.268, 6.569)	0.729
	Age	1 year	0.648	(0.525, 0.799)	<0.001
	Group: deaf	koda	0.386	(0.217, 0.687)	0.001
	Group: DDCI	koda	0.613	(0.316, 1.192)	0.151
	Complexity: handshape	increment of 1	1.015	(0.782, 1.317)	0.913
	Complexity: 2-handed	1-handed	0.625	(0.360, 1.084)	0.094
	Complexity: 3 SM	two or no	1.896	(0.961, 3.744)	0.065
	Complexity: mseq	no seq	7.404	(3.107, 17.642)	<0.001
